# Determination of stress-induced degradation products of cetirizine dihydrochloride by a stability-indicating RP-HPLC method

**DOI:** 10.1186/s40199-014-0082-5

**Published:** 2014-12-09

**Authors:** Paloma Flórez Borges, Pilar Pérez Lozano, Encarna García Montoya, Montserrat Miñarro, Josep R Ticó, Enric Jo, Josep M Suñe Negre

**Affiliations:** Pharmacy and Pharmaceutical Technology Department, Faculty of Pharmacy, University of Barcelona, Avda Joan XXIII s/n 08028, Barcelona, Spain; Reig Jofre Group, c. Gran Capitá 6 08970, Sant Joan Despi, Barcelona, Spain

## Abstract

**Background:**

A new, simple and accurate stability-indicating reverse phase high performance liquid chromatography method was developed and validated during the early stage of drug development of an oral lyophilizate dosage form of cetirizine dihydrochloride.

**Methods:**

For RP-HPLC analysis it was used an Eclipse XDB C8 column 150 mm × 4.6 mm, 5 μm (Agilent columns, Barcelona, Spain) as the stationary phase with a mobile phase consisted of a mixture of 0.2 M K_2_HPO4 pH 7.00 and acetonitrile (65:35, v/v) at a flow rate of 1 mL min ^−1^. Detection was performed at 230 nm using diode array detector. The method was validated in accordance with ICH guidelines with respect to linearity, accuracy, precision, specificity, limit of detection and quantification.

**Results:**

The method results in excellent separation between the drug substance and its stress-induced degradation products. The peak purity factor is >950 for the drug substance after all types of stress, which confirms the complete separation of the drug substance peak from its stress induced degradation products.

Regression analysis showed r^2^ > 0.999 for cetirizine dihydrochloride in the concentration range of 650 μg mL ^−1^ to 350 μg mL^−1^ for drug substance assay and a r^2^ > 0.999 in the concentration range of 0.25 μg mL^−1^ to 5 μg mL^−1^ for degradation products. The method presents a limit of detection of 0.056 μg mL ^−1^ and a limit of quantification of 0.25 μg mL^−1^. The obtained results for precision and accuracy for drug substance and degradation products are within the specifications established for the validation of the method.

**Conclusions:**

The proposed stability-indicating method developed in the early phase of drug development proved to be a simple, sensitive, accurate, precise, reproducible and therefore useful for the following stages of the cetirizine dihydrochloride oral lyophilizate dosage form development.

## Background

**Figure 1:**
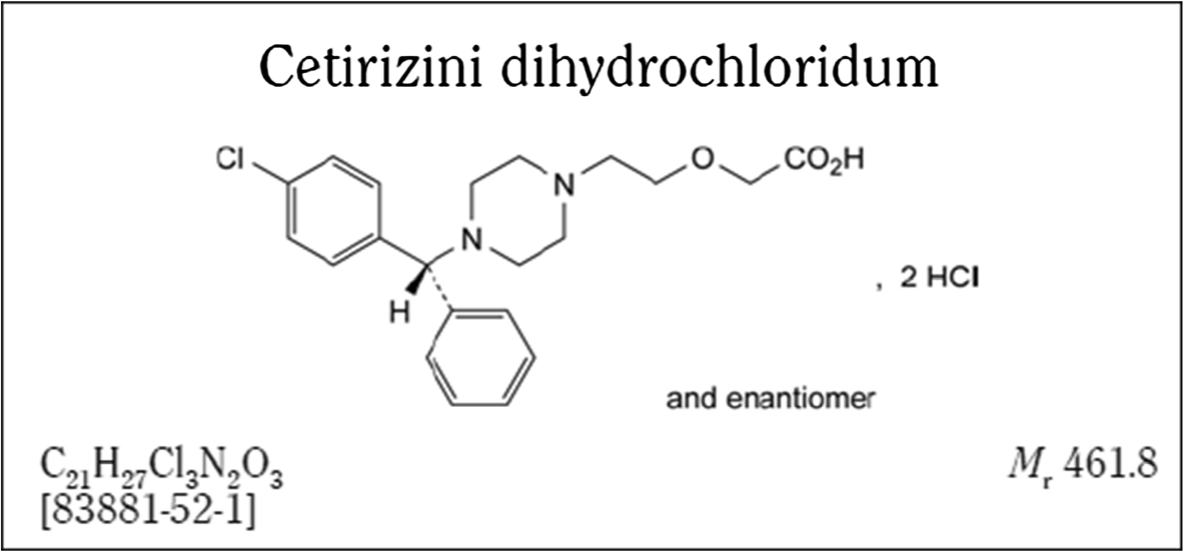
**Chemical structure of cetirizine dihydrochloride.** Ph. Eur. 7th Edition 2014 (8.0).

In the early stage of drug development, forced degradation studies are used to facilitate the development of an analytical methodology, in order to obtain a better understanding of the drug substance (DS) studied and the final drug product (DP) stability, providing data regarding degradation pathways and degradation products (DE) [1]. Such studies are needed to assure that all the regulatory requirements of a drug are fulfilled, such as the identification of possible DE, degradation pathways and intrinsic stability of the drug molecule. Part of the study is the development and validation of the stability indicating analytical method involved [2,3]. The overall objective of this work is to develop a new formulation with the drug substance (C_21_H_27_Cl_3_N_2_O_3_) cetirizine dihydrocloride (CTZ; the dihydrochloride of a 2-[4-chlorobenzhydryl) piperazin-1-yl] ethoxyacetic acid). CTZ is a non-sedative H_1_ antihistaminic drug, a piperazine derivative and metabolite of hydroxyzine (Figure 1) [4]. CTZ presents an increased degree of polarity, which makes it less capable of crossing the blood brain barrier, hence reducing the sedative side effects in comparison with first generation antihistamines, such as diphenhydramine and hydroxyzine [5–7]. CTZ is administrated generally in tablets and liquid forms orally to promote the relief of symptoms related to allergic rhinitis, chronic idiopathic urticaria and other rashes [8,9].

This new formulation consists of an oral lyophilized dosage form, whose aim is to facilitate swallowing (in the case of patients with dysphagia, such as children and elderly, for instance), easy to administer, effective, safe and stable over time.

Several HPLC methods have been reported in literature for the determination of CTZ alone [10–13] and also determining CTZ simultaneously with other drug substances, as in multicomponents preparations [14–16]. In order to develop a new chromatographic method for the determination and quantification of CTZ and its DE generated after a forced degradation study, several chromatographic methods for CTZ were investigated in the literature. Among them, was the Ph. Eur. method for CTZ [17]. However, the latter was discarded due to the use of a normal phase chromatographic column and mobile phase that used much organic solvent (acetonitrile, not very cost-effective). Also, the Ph. Eur. method presents a very acid mobile phase pH (pH <0.5), which is known to diminish the life span of the chromatographic column [18]. Also, some chromatographic analytical methods [12,13] used chromatographic columns of reverse phase, usually C18 and C8. Depending on the type of separation pursued (as for instance, CTZ combined with another DS), isocratic or gradient methods were used, and also mobile phases with ionic pairing. We have developed a reverse-phase high performance liquid chromatography (RP-HPLC) method by studying the effect of the stationary phase (C18 or C8 analytical columns) on peak resolution, the influence of pH -mobile phase- when adjusting the desired retention time (t_**R**_) for the DS. Plus, by using a reverse-phase column, we reduced the amount of organic solvent (acetonitrile) used for the identification of the DS, in comparison to the analytical method validated by Ph. Eur. [17], which uses a normal phase chromatographic column, requiring more organic solvent due to its characteristics [18–25]. Therefore the aim of this study is to determine all possible DE generated under stress conditions, by developing and validating a stability-indicating RP-HPLC method for cetirizine dihydrochloride in the early stage of drug development of an oral lyophilizate.

## Methods

### Chemicals and reagents

All chemicals were analytical grade and used as received. All solutions were prepared in Milli-Q deionized water from a Milli Q gradient A10 water purification system (Molsheim, France). CTZ bulk powder (Cetirizine dihydrochloride, Ph. Eur) was purchased from Jubilant Lifesciences Ltd (Mysore, India) and kindly provided by Reig Jofre Group (Barcelona, Spain). HPLC grade acetonitrile was obtained from Panreac (Barcelona, Spain). Ortho-phosphoric acid 85% was purchased from Panreac (Barcelona, Spain). Potassium phosphate dibasic Ph. Eur. (K_2_HPO_4_) was purchased from Fagron (Terrassa, Spain). Hydrochloric acid 37%, sodium hydroxide and hydrogen peroxide (H_2_O_2_) at 33% were purchased from Panreac (Barcelona, Spain).

### Equipment and chromatographic conditions

Samples were analyzed on Dionex Ultimate 3000 HPLC Thermo Fisher Scientific (California, USA), equipped with data system Chromeleon version 6.8 SP2 Build 2284, with degasifier SR3000, LPG-3400 quaternary pump, injector WPS3000, oven 6P TCC-3100, UV–vis detector PDA-3000. For initial development studies it was used an analytical chromatographic column Kromasyl 100-5C18 150 mm × 4.6 mm, 5 μm particle size (Tecnokroma Akzonobel, Terrasa, Spain). For final development and method validation, it was used an analytical chromatographic column Eclipse XDB-C8 150 mm × 4.6 mm, 5 μm particle size (Agilent columns, Barcelona, Spain). An isocratic mobile phase consisting of acetonitrile and 0.2 M potassium phosphate dibasic Ph. Eur. buffer solution at pH 7.00 (35:65 v/v) was used, and the analysis was carried out at a flow rate of 1 mL min ^−1^. All determinations were performed at 30°C. The injection volume was 25 μL. The detector was set at λ 230 nm. The peak homogeneity was expressed in terms of peak purity factor and was obtained directly from spectral analysis report using the above mentioned software. Other apparatus included a Crison micropH 2002 pH meter (Barcelona, Spain) and Heraeus oven T5028 for thermal degradation (dry heat at 105°C) (Hanaus, Germany).

### Forced degradation studies and preparation of samples

The forced degradation studies were carried out by preparing several standard solutions of CTZ at 500 μg mL^−1^, for each degradation study. Each sample was analyzed according to the previous procedures described under the proposed analytical method. In order to determine whether the analytical method is suitable to be a stability-indicating assay, forced degradation studies under different conditions were carried out according to the following procedure:

- Acid and basic hydrolysis: 5 mg of bulk powder was treated with 5 mL of 0.1 M HCl and 0.1 M NaOH. The flasks were placed in a dry air oven at 105°C. Another 5 mg of bulk powder was also treated with 5 mL of 0.1 M HCl and 0.1 M NaOH at room temperature, for 24 hours.
- Oxidation with H_2_O_2_ at 33%: 5 mg of bulk powder was exposed to 5 mL of hydrogen peroxide at 33% (W/v). The vial was kept at room temperature for 24 hours.
- Infrared (IR) and Ultraviolet (UV) light: 5 mg of bulk powder was exposed under an infrared lamp and another 5 mg of bulk powder was exposed under an ultraviolet lamp, for 24 hours.
- Humidity HR 79%: the 5 mg bulk powder sample was placed inside a humidifier with HR 79%, for 24 hours.
- Heat at 105°C: 5 mg of bulk powder sample was placed inside a 105°C dry air oven for 24 hours.
- Shed sunlight for 15 days: 5 mg of bulk powder was kept in a vial for 15 days, at room temperature and exposed to direct sunlight.

Once the stress conditions were complete, 10 mL of 0.2 M phosphate buffer (pH 7.00) was added to the samples in order to achieve the standard solution concentration of 500 μm mL-1. Moreover, all the solutions and blanks were filtered with a 0.45 μm syringe filtration disk PVDF. Results were compiled in terms of relative retention times (rtR) found during the analysis.

### Validation of the analytical method

In order to validate the RP-HPLC method developed, ICHQ2B guideline recommendations were followed, in terms of selectivity, linearity, range, accuracy, precision, limit of detection (LOD) and limit of quantification (LOQ) [26]. In order to fulfill ICH specifications in terms of linearity and range for the analytical method (content uniformity and assay of DS and finished product), a linear range within 70-130% was studied, by analyzing a series of three replicates, i.e., three independent sets (k = 3), each with seven different concentrations (n = 6): 350 μg ml ^−1^ - 650 μg ml ^−1^, considering 500 μg ml ^−1^ as 100% (standard solution), in order to provide information on the variation in peak area values between samples of the same concentration. For evaluation of the precision estimates, repeatability and intermediate precision were performed at three concentration levels (650, 500 and 350 μg ml ^−1^, corresponding to 130, 100 and 70%), and 10 injections of each sample (K = 10), per day. Mean average, standard deviation (SD) and relative standard deviation (RSD) of t_R_ and the peak area achieved individually of day 1 and 2 were calculated. After the HPLC analysis, the response factor (RF) was calculated between the response (Y) and concentration achieved (X), as Y/X. Therefore, mean average, SD and RSD were calculated using the response factors obtained with an Excel 2007 spread sheet. The response factors results must comply with a RSD ± 2%. For accuracy the concentration found expressed by function of repeatability of the standard solution, relative error in percentage and the percentage of recovery, with mean average, SD and RSD deviation of each of the three concentrations studied (650, 500 and 350 μg ml ^−1^, was considered of three replicates) were calculated. For the DS, 98-102% percentage of recovery was considered as being acceptable [27].

For the determination and quantification of the DE, linearity, precision, accuracy and LOD and LOQ were calculated. In order to carry out this validation, further dilutions from a stock solution of 500 μg ml ^−1^ with the specified mobile phase were carried out in order to achieve the correspondent concentrations: 5 μg mL^−1^, 2.5 μg mL ^−1^, 1.25 μg mL ^−1^, 0.5 μg mL^−1^, 0.25 μg mL^−1^, 0.125 μg mL ^−1^. A total of seven independent calibration curves, i.e., seven replicates ( k = 7) were prepared. The LOD and LOQ were calculated by the ratio between the standard deviation of y-intercepts of regression lines of the seven calibration curves mentioned before by averaging the slopes of calibration curve multiplied by 3.3 and 10, respectively [26,27]. Each serial dilution (k = 7) was analyzed, with n = 6 (level of concentrations).

In terms of relative error and percentage of recovery three concentrations (5, 1.25 and 0.25 μg mL^−1^) from the range of DE were evaluated. All the solutions prepared were filtered with a 0.45 μm syringe filtration disk PVDF to the vials for injection in the HPLC system.

## Results and discussion

### HPLC method development

As an early stage study of drug development, our goal was to acknowledge all possible DE generated under stress conditions for CTZ. The information acquired in the early stage of the study will lead us to a better understanding of the DS itself and also the possible DE that we may find during the next step of the oral lyophilized development study. Therefore it was not our objective the development of a fast analytical method for the DS per se, but actually the development of an analytical method that could detect a complete profile of DE for this DS, leaving for the following studies of drug development the aim of reducing run time, for instance.

**Figure 2:**
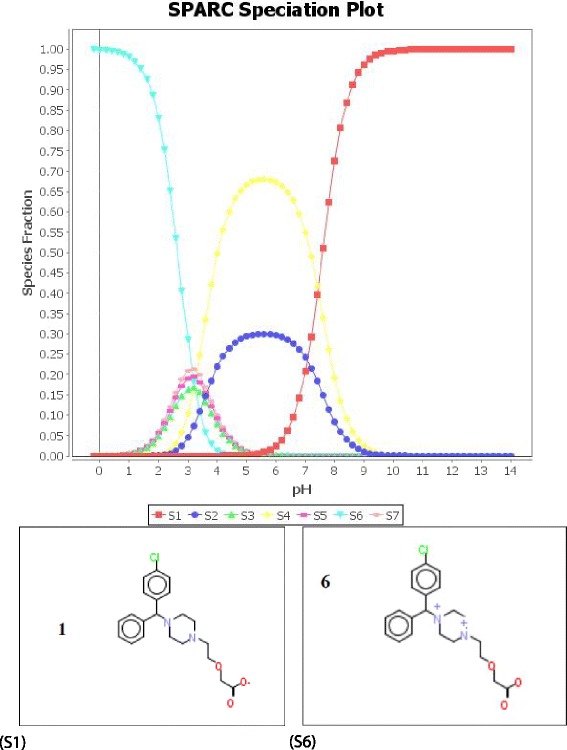
**SPARC speciation plot for cetirizine dihydrochloride.** Anionized (S1) and protonized (S6) species.

**Figure 3:**
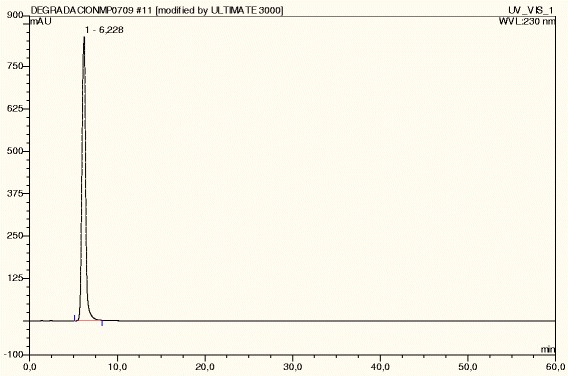
**Cetirizine dihydrochloride under normal conditions.**

CTZ is freely soluble in water, and practically insoluble in acetone and metilen chlorate [17]. Considering its hydrophilic nature, reverse phase columns were chosen in order to investigate the chromatographic profile with two types of packing material for stationary phases: C18 (more hydrophobic, octadecylsilyl), and C8 (intermediate hydrophobicity, octylsilyl). We also studied the molecule of CTZ using the physicochemical calculator SPARC (Sparc Performs Automated Reasoning in Chemistry) developed by the United States Environmental Protection Agency (EPA) for the purpose of predicting which pH would suit best for CTZ ionization [28]. The speciation plot for CTZ (Figure 2) shows that pH3 is not recommended for the ionization of CTZ due to the existence of six different species of ionized CTZ with no clear definition among them, whereas at pH2 we can find the protonated CTZ (S6) and at pH7 the anionized CTZ (S1) (Figure 3). The pKa values estimated for CTZ are: 2.7 (pKa1), 3.6 (pKa2) and 7.6 (pKa 3) [29]. However, due to the finding of chromatographic methods that used buffer solutions at pH3 or around 3 (2.8, 3.5, for instance) we decided to considered pH3 in our study [10–12,14]. During the preliminary studies of the analytical method development, we have combined different proportions of acetonitrile and aqueous solution at pH3 (MilliQ water acidified at pH3 with orto-phosphoric acid 85%). The preliminary studies were carried out by the injection of a 500 μg mL ^−1^ solution of CTZ, using a C18 analytical chromatographic column, flow rate of mobile phase of 1 mL min ^−1^, an injection volume of 25 μL, oven temperature of 30°C, 230 nm of wavelength, in isocratic mode. The effects of the optimum eluent composition were studied, obtaining a retention time (t_**R**_) of eight minutes for CTZ with a 35:65 (v/v) of acetonitrile and aqueous solution at pH3. Also, we tried to adjust the mobile phase –studying the effect of the proportion between the organic solvent and buffer solution pH– in order to achieve a t_**R**_ of approximately 6–7 for CTZ. To diminish the t_**R**_, we finally tried to use a buffer solution of 0.2 M K_2_HPO_4_ pH7. We observed that maintaining the same proportions of organic solvent and buffer solution, but changing the pH from 3 to 7, using potassium phosphate dibasic buffer solution at 0.2 M solution at pH7, we achieved a t_**R**_ of 5–6 minutes. However, CTZ peak shape presented tail broadening. Therefore, in order to avoid using ion pair to improve peak resolution, we have changed the analytical chromatographic column C18 (Kromasyl 100–5 C18) to a C8 (Eclipse XDB C8), and tested with the same conditions as before: acetonitrile and phosphate buffer solution pH 7 (35:65, respectively), injection volume 25 μL, flow rate 1 mL min ^−1^, temperature 30°C, 230 nm of wavelength. This resulted in eliminating tail broadening. Having determined the eluent proportions of the isocratic mobile phase and the pH of the aqueous solution as pH7, obtaining the desired t_**R**_, we have established the stability-indicating method to carry out the forced degradation studies.

### Results of forced degradation study

**Figure 4:**
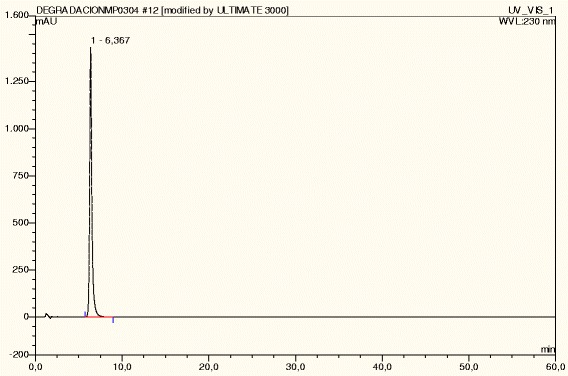
**Cetirizine dihydrochloride under humidity HR79%.**

**Figure 5:**
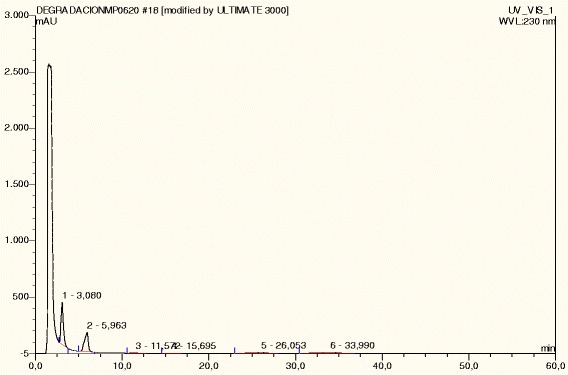
**Cetirizine dihydrochloride under H**_**2**_ **O**_**2**_ **at 33%.**

**Figure 6:**
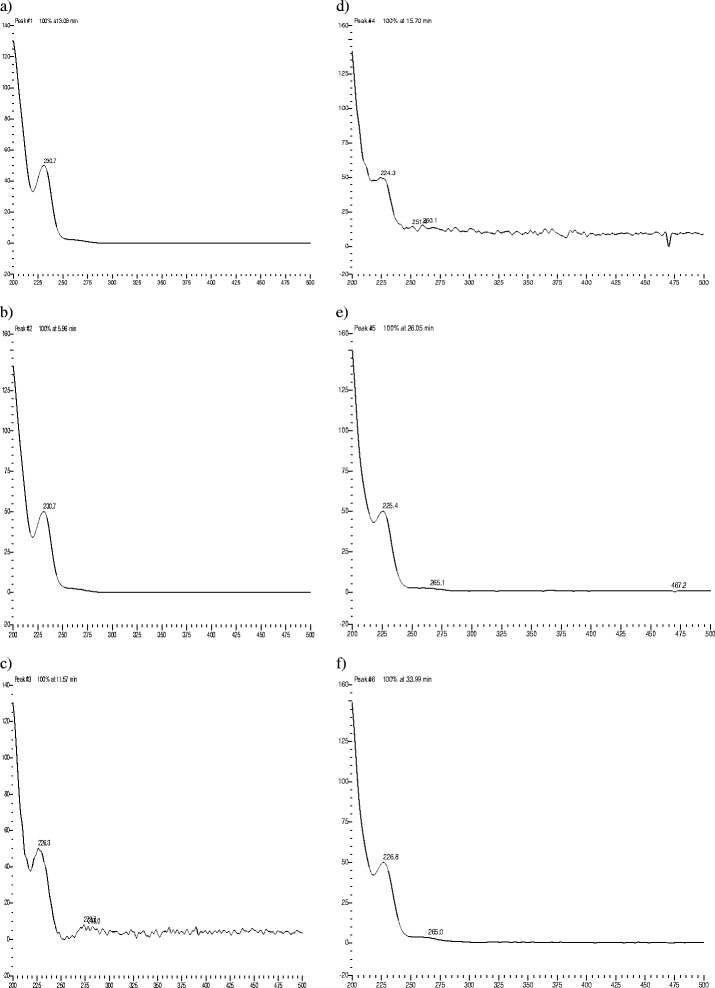
**Chromatographic spectra of cetirizine dihydrochloride (b) and its degradation products (a, c, d, e and f) under H**_**2**_ **O**_**2**_ **at 33%, in nm.**

CTZ was degraded up to 19% under acid hydrolysis at 105°C, presenting five degradation peaks. Under basic hydrolysis at 105°C, CTZ was degraded up to 15%, presenting twelve degradation peaks, followed by shed sun light (10%, one degradation peak), UV (9%, four degradation peaks), IR (8%, four degradation peaks) and dry heat at 105°C (3%, 6 degradation peaks). CTZ under photolytic stress -shed sunlight during 15 days, IR and UV light- presented degradation peaks with two, four and five peaks, respectively (Table 1). CTZ presented DE peaks under acid (three DE peaks) and basic hydrolysis at room temperature (three DE peaks). However, degradation was not substantial in both cases (Table 2). Comparing the chromatographic profile of CTZ dissolved in buffer solution (Figure 3) with no stress conditions (normal conditions) with CTZ under Humidity HR79% (Figure 4), it is observed that CTZ showed no substantial degradation under Humidity HR79%, presenting a similar chromatographic profile with CTZ dissolved in buffer solution at the same concentration. Under oxidative stress, CTZ presented 79% of degradation, showing five degradation peaks (Figure 5) and in Figure 6 it can be visualized by the chromatographic spectra of each DE and CTZ. Furthermore, the peak purity value for CTZ under oxidative stress was of 998 (considering 1000 as 100% match), indicating a homogenous peak (Table 2). However, in the beginning of the elution process, the diode-array assay detects DE higher that its threshold, demonstrating a possible saturation of the chromatographic column with H_2_O_2_ at 33%. There is also the hypothesis that 79% of decomposition can also be the result of the degradation of DE, which would generate more DE, due to the exposure of CTZ during 24 hours under oxidative condition. This leads to the conclusion that may be necessary to change the oxidative stress condition procedure, by reducing the concentration of peroxide (33%) or reducing the time of exposure of the DS with H_2_O_2_ (24 hours), or both. Satisfactory results were obtained studying the peak purity index for CTZ under stress conditions, which confirms the high specificity of the analytical method for CTZ (Table 2).

**Table 1:** **Summary of product degradation peaks in relative retention time (**rt_**R**_ **)**

**Stress conditions**	**rt**_**R**_ **(min)**								**CTZ**										
Humidity HR79%									1.00										
Acid hydrolysis *RT			0.51				0.81	0.87	1.00										
Acid hydrolysis at 105°C	0.46		0.50		0.64			0.87	1.00								5.00		
Ultraviolet light (UV)			0.52	0.59			0.81		1.00		2.00				4.30				
Infrared light (IR)			0.51	0.60	0.66		0.81		1.00										
Basic hydrolysis *RT			0.53				0.81	0.87	1.00										
Basic hydrolysis at 105°C	0.46	0.48	0.51	0.58		0.71		0.86	1.00	1.60	2.00		2.80	3.40			5.00		9.10
Dry heat at 105°C			0.50			0.72	0.80	0.90	1.00						4.30		5.00		
Shed sunlight 15 days				0.60					1.00										
H_2_O_2_ at 33%			0.51						1.00		1.90	2.60				4.40		5.70	
Normal conditions									1.00										

*RT: Room temperature.

**Table 2:** **Peak purity determination by diode-array UV–vis spectra of CTZ and stress studies results**

**Forced degradation conditions**	^**a**^ **Peak purity index match**	**Decomposition (%)**	**Extent of decomposition**
Humidity HR79%	952	0	None
Acid hydrolysis *RT	990	0	None
Acid hydrolysis at 105°C	998	19	Substantial
Ultraviolet light (UV)	962	9	Substantial
Infrared light (IR)	962	8	Substantial
Basic hydrolysis *RT	972	0	None
Basic hydrolysis at 105°C	986	15	Substantial
Dry heat at 105°C	953	3	Substantial
Shed sunlight 15 days	996	10	Substantial
Oxidative medium *RT	998	79	Substantial
CTZ (phosphate buffer solution)	990	0	Normal conditions

^a^indicates the value for peak purity index match of CTZ above 950, considering 1000 as 100% match; *RT: Room temperature.

### Method validation

The developed method was validated using ICH guidelines [26]. Validation parameters included linearity, precision, accuracy, precision, and specificity, LOD and LOQ [26,27].

### Assay for drug substance method

Linearity for CTZ assay was verified by triplicate analysis of seven different concentrations, i.e., three sets of 130-70% range of CTZ. As a result, the linear regression equation was found to be Y = 769.56 X + 14.573 (r^2^ = 0.9994, k = 3 (number of replicates), n = 7 (level of concentrations), 650 μg mL ^−1^ to 350 μg mL ^−1^) for CTZ. In which, Y was the dependent variable, X was independent variable, 769.56 was the slope and which showed change in dependent (Y) variable per unit change in independent (X) variable; 14.573 was the Y-intercept i.e., the value of Y variable when X = 0.

As for the analytical method precision (Table 3), three concentration levels (650, 500 and 350 μg ml ^−1^, corresponding to 130, 100 and 70%), and 10 injections of each sample, per day were prepared. The results have shown the repeatability and intermediate precision presenting a RSD inferior to 2.7% according to AOAC [27] (1.43% and 1.03%, respectively). In terms of accuracy, according to the obtained results (Table 4), the percentage of recovery ranges from 98.56 to 101.44, from being within the limits established according to AOAC [27] (98-102%), which indicates the accuracy of the method for CTZ.

**Table 3:** **Repeatability and intermediate precision according to retention time (t**_**R**_ **) and peak area for CTZ assay**

	**t**_**R**_			**Peak area**	
**Day**	**μg mL** ^**−1**^	**Mean average ±** ^**a**^ **SD (min)** ^**b**^ **K = 10**	^**c**^ **RSD (%)**	**Mean average ±** ^**a**^ **SD (mAU.min)** ^**b**^ **K = 10**	^**c**^ **RSD (%)**
1	650	5.79 ± 0.0274	0.4747	512.54 ± 1.7865	0.5365
	500	5.79 ± 0.0327	0.5646	399.69 ± 1.9090	0.4777
	350	5.80 ± 0.0228	0.3929	281.21 ± 1.7867	0.6353
2	650	5.77 ± 0.0195	0.3384	511.75 ± 2.2059	0.4310
	500	5.78 ± 0.0149	0.2584	399.54 ± 1.5972	0.3997
	350	5.80 ± 0.0248	0.4279	281.63 ± 1.0483	0.3722

^a^SD (Standard deviation); ^b^K (number of injections); ^c^RSD (Relative standard deviation).

**Table 4:** **Concentrations found, relative error in percentage, percentage of recovery and estimates for CTZ assay**

^**a**^ **Theoretical concentration (μg mL** ^**−1**^ **)**	**Concentration found (μg mL** ^**−1**^ **)**	**Relative error%**	^**b**^ **Recovery%**	**Mean recovery**	^**c**^ **SD of recovery**
650	641.29	1.25	98.75	98.66	0.09
650	641.27	1.36	98.65		
650	640.67	1.45	98.56		
500	500.96	0.19	100.19	100.49	0.57
500	500.71	0.14	100.14		
500	505.80	1.14	101.16		
350	352.16	0.61	100.61	100.95	0.43
350	352.77	0.78	100.79		
350	355.05	1.42	101.44		

^a^650 μg mL^−1^ = 130%, 500 μg mL^−1^ = 100%, 350 μg mL^−1^ = 70%; ^b^recovery limits (98-102%); ^c^SD (Standard deviation).

It was studied seven independent sets of dilutions, i.e., seven replicates (k =7), each set with six different concentrations (n = 6) in the range of 5–0.125 μg mL^−1^. Calculating LOD and LOQ by the ratio between the SD of y-intercepts of regression lines of the seven serial dilutions of six different concentrations mentioned before by averaging the slopes of calibration curve multiplied by 3.3 and 10, respectively, the analytical method presented a LOD of 0.056 μg mL^−1^ and a LOQ of 0.17 μg mL ^−1^. However, it was demonstrated that the LOQ value of 0.17 μg mL ^−1^ was not lineal. Therefore, taken into account the range defined in ICH guidelines to the DE, for linearity reasons it was considered the range around a suggested (probable) limit [26]. Therefore, the linearity should be established from the LOQ to 120%. So a new LOQ (SD (μg mL ^−1^) = 0.0111, RSD (%) = 6.1605) was established (0.25 μg mL ^−1^). The linear regression equation was found to be Y = 0.8125X-0.014 (r^2^ = 0.9999, n = 5 (level of concentrations), k = 7 (number of replicates), 0.25-5 μg mL^−1^) (Table 5).

**Table 5:** **Mean average, standard deviation (SD) and relative standard deviation (RSD%) of peak area mAU (5 – 0.125 μ mL** ^**−1**^ **)**

**Theoretical concentration (μg mL** ^**−1**^ **)**	**Mean concentration average ±** ^**a**^ **SD (μg mL** ^**−1**^ **) (** ^**b**^ **k = 7)**	^**c**^ **RSD (%)**
5.000	4.0495 ± 0.1040	2.5701
2.500	2.0121 ± 0.0251	1.2502
1.250	1.0012 ± 0.0326	3.2622
0.500	0.4021 ± 0.0327	8.1334
0.250	0.1818 ± 0.0111	6.1605

^a^SD (Standard deviation); ^b^k (number of replicates); ^c^RSD (relative standard deviation).

In reference to the results of the analytical method validation, in both Table 5 (repeatability) and Table 6 (recovery), the RSD and the range complies with RSD permitted (2.7%) and the range (90-110%) according to AOAC [27], assuring the applicability of the developed analytical method for the determination and quantification of DE.

**Table 6:** **Relative error (%) and percentage of recovery**

**Concentration (μg mL ** ^**−1**^ **)**	**Mean concentration found (μg mL** ^**−1**^ **) ( ** ^**a**^ **k = 7)**	^**b**^ **Relative error% (mean)**	^**c**^ **Mean Recovery%**	^**d**^ **SD of recovery%**
5	5.000	0.620	100.070	0.74
1.25	1.248	3.310	99.840	4.10
0.25	0.235	9.390	94.210	10.00

^a^k = 7 (number of replicates); ^b^mean relative error% ; ^c^mean recovery%; ^d^standard deviation.

## Conclusions

A new and simple RP-HPLC method was developed for the determination of CTZ and its DE during the early stage of drug development of an oral lyophilized dosage form. The proposed method was demonstrated to be linear, precise, accurate and specific, based on method validation. Satisfactory results were obtained in separating the peak of CTZ from the DE produced by forced degradation. Plus, it is a cost-effective method that requires a simple mobile phase (phosphate buffer solution and acetonitrile, 65:35 v/v) and also does not require the use of ion pairing, which can result in difficulty in recovering initial column properties. It was also able to separate with good specificity the DS peak from the entire DE generated during the stress condition study, which help us in the next step of the drug development of the oral lyophilizate, by adapting the validated method considering further aspects, such as the interactions between CTZ and the excipients chosen for the final medicinal product. The proposed analytical method proved to be stability-indicating and therefore useful in the following stages of drug development.

## Abbreviations

DS: Drug substance
DP: Drug product
DE: Degradation products
CTZ: Cetirizine dihydrochloride
RP-HPLC: Reverse phase high performance liquid chromatography
t_**R**_: Retention time
rt_**R**_: Relative retention time
LOD: Limit of detection
LOQ: Limit of quantification
IR: Infrared light
UV: Ultraviolet light
